# Mixture composition design of magnesium oxychloride cement-stabilized crushed stone materials applied as a pavement base

**DOI:** 10.1038/s41598-024-62602-1

**Published:** 2024-05-22

**Authors:** Huzhu Zhang, Aolin Luo, Lijuan Sun

**Affiliations:** School of Transportation Science and Engineering, Jilin Jianzhu University, Changchun, People’s Republic of China

**Keywords:** Magnesium chloroxylate cement stabilized crushed stone, Pavement subgrade, Proportion design, Unconfined compressive strength, Water resistance enhancement method, Response surface method, Engineering, Materials science

## Abstract

Conventional binding materials, such as silicate cement and lime, present high energy consumption, pollution, and carbon emissions. Therefore, we utilize crushed stone as a stabilization material. Magnesium oxychloride cement (MOC) is modified and used as an inorganic admixture owing to its eco-friendly nature and low carbon content. We analysed the control indicators of an integrated design of MOC-stabilized crushed stone by conducting unconfined compressive strength and water-resistance tests. The optimum mixing composition of the MOC-stabilized crushed stone was determined through the response surface methodology. We determined the best approach and dosage for improving the water resistance of MOC-stabilized crushed stone by comparing the effects of four modification methods: fly ash, citric acid + silica fume, phosphoric acid + waterborne polyurethane, and dihydrogen phosphate potassium salt. We also perform a comparison with 5% ordinary silicate cement-stabilized crushed stone. The results indicate that the MOC-stabilized crushed stone exhibits a rapid increase in strength in the early stage, but this rate reduces after 28 days. The mixing design employs the 4-day unconfined compressive strength and 1-day water resistance coefficient as the technical indicators. The best mixing composition includes a 4.27% MOC dosage and a molar ratio of MgO/MgCl_2_ of 5.85. We use 1% citric acid + 10% silica fume in equal amounts to replace the MOC dopant method for composite modification of the MOC stabilized crushed stone. Consequently, the 1-day water resistance coefficient before water immersion is significantly increased from 0.78 to 0.91 and its 4-day unconfined compressive strength is only reduced by 0.10 MPa. This significantly improves the water resistance of the MOC-stabilized crushed stone and ensures that its strength remains unaffected, which is the optimal modification method. However, this method must ensure that a small amount of citric acid and silica fume are uniformly distributed in the MOC-stabilized crushed stone, which increases the construction difficulty of the road base.

## Introduction

Typically, inorganic binding materials such as silicate cement and lime are used for the construction of road transportation infrastructure. However, these materials consume a large amount of non-renewable resources during the production and preparation process, emitting a large amount of carbon dioxide and causing severe environmental pollution. Magnesium oxychloride cement (MOC) is an air-hardened cementitious material formed by mixing light-fired magnesium oxide powder with magnesium chloride solution. MOC presents the advantages of high strength, good abrasion resistance, resistance to high and low temperatures, low weight, and corrosion resistance. The calcination temperature of light-fired MgO is much lower than that of silicate cement clinker and lime. Furthermore, the magnesium chloride solution is processed using seawater or salt by-products obtained during salt production in salt lakes. Therefore, MOC cement is greener, presents low carbon emissions, and consumes less energy than the conventional inorganic bonding materials, such as ordinary silicate cement and lime. However, it exhibits poor water resistance and is unsuitable for use in humid environments since it is an air-hardened cementitious material, which significantly limits its application in road engineering. Therefore, on the basis of the existing application and research of magnesium chloride cement, to explore the proportion design method, optimal proportion and water resistance enhancement method of magnesium chloride cement stabilized gravel material, in view of the performance characteristics and requirements of the materials used in pavement grass-roots level, so as to innovate and introduce it into the field of stabilizing the grass-roots level of the road surface, which has important scientific value and practical significance for the advancement of energy conservation and emission reduction in the field of transportation infrastructure construction, sustainable development of the road construction, as well as the realization of the national "double carbon" strategy.

The existing research on MOC is primarily focused on their performance-influencing factors, modification methods, and engineering applications. The MgO/H_2_O/MgCl_2_ molar ratio, hydration products, temperature, and humidity during curing are the main factors affecting the MOC properties. The 5-phase crystals produced through hydration are the main source of MOC strength, and a suitable MgO/H_2_O/MgCl_2_ molar ratio can increase the number of 5-phase crystals that are produced, thereby improving the strength of the MOC. Qiao et al. reported that the MgO/MgCl_2_ molar ratio is the main factor affecting the long-term strength of MgCl_2_ cement concrete, and MOC concrete presents the best performance with an MgO/MgCl_2_ molar ratio of 5.4^1^. Li reported that the molar ratio of MgO/MgCl_2_ was the most favourable for the generation of 5-phase crystals when the ratio of MgO/MgCl_2_ was 11–17 and the ratio of H_2_O/MgCl_2_ was 12–18^2^. Wang et al. reported that the effect of the MgO/MgCl_2_ molar ratio on the strength of MOC straw boards was the most favourable for the generation of 5-phase crystals when the ratio of MgO/MgCl_2_ was 7^3^. The amount of water blending also affects the performance of the MOC: an excessively large molar ratio of H_2_O/MgCl_2_ produces a large number of loose magnesite structures, which affects the strength of the MOC, and an excessively small molar ratio of H_2_O/MgCl_2_ results in the solid MgO and MgCl_2_ not being effectively involved in the hydration reaction. Liu et al. reported that the optimum molar ratio of H_2_O/MgCl_2_ is 15–20 when the molar ratio of MgO/MgCl_2_ was 3–4; this presents a better MOC hydration reaction and the main hydration products were 5-phase and 3-phase with the best mechanical properties^4^. If the MOC curing process temperature is too high, it may cause the decomposition of the 3-phase and 5-phase crystals or cause deformation and cracking in the MOC specimen due to uneven heat dissipation. Conversely, if this temperature is too low, it reduces the rate of the hydration reaction, resulting in incomplete hydration. Aiken et al. reported that the curing temperature of the MOC is best stabilized in humid environments at 20 °C to 50°C^5^.

Despite the excellent mechanical properties of the MOC in terms of strength, it has poor water resistance, and the 3-phase and 5-phase crystals typically decompose into Mg(OH)_2_ in humid environments, which significantly reduce the compressive strength^6,7^. Several modification studies have been conducted on enhancing the water resistance of the MOC. Active mineral admixtures such as silica fume and fly ash can optimize the porous structure of the MOC, thus improving its density, compressive strength, and water resistance^8–13^. For example, phosphoric acid, citric acid, hydroxyacetic acid, tartaric acid, and soluble phosphate can form a gel 5-phase crystal with low crystallinity, along with chelate by coordinating between the acid group therein and Mg^2+^, effectively preventing the transformation of MOC hydration products from the 5-phase crystals to the 3-phase crystals. This helps in forming a more compact spatial mesh structure and significantly enhances the water resistance of MOC^14–18^. Some studies have reported that polymers or surfactants, such as phenylpropylene emulsions, waterborne polyurethane, tetraethoxysilane, and other organic chemicals, can enhance water resistance by reducing the contact between the internal hydration products and water by generating polymer waterproof films or hydrophobic layers in the MOC^19–21^.

MOC is widely used as a fireproof or decorative material owing to its high strength, high and low-temperature resistance, and low weight^22,23^. However, it is not widely applied in the field of pavement subgrades due to its limited water resistance. Extensive research has been conducted to overcome this issue. Wang et al. used the MOC as a curing agent and fly ash as a modifier to cure silt and reported that MOC could significantly improve the compaction and mechanical properties of the soil^24^. Zheng Weixin et al. employed MOC to build road pavements in Northwest China and reported that its compressive strength decreased in flexural tensile strength after two rainy seasons; they also observed through XRD and scanning electron microscopy (SEM), that the 5-phase hydrolysis caused by rainwater soaking was the main reason for the decrease in the pavement strength^25^. Huang Qing et al. reported that the MOC mortar exhibits excellent salt resistance after saltwater immersion through XRD, thermogravimetric analysis, and scanning electron microscopy (SEM), which can be applied to saline soil and salt lake areas^26^. Sun Yi performed an experimental analysis on the road performance of a magnesium cement-stabilized crushed stone mix and observed that its mechanical indices met the requirements for the use of a pavement base after modification by fly ash, but the water resistance performance was still poor^27^.

In summary, both domestic and international researchers have achieved significant results in the reasonable proportioning of MOC, performance improvement methods, and their application in engineering practice, and have gradually attempted to implement it in the field of soil reinforcement and road engineering. However, limited research has been conducted on implementing MOC as an inorganic binding material in road pavement engineering, and the material itself faces the drawback of insufficient water resistance. Furthermore, limited information is available for reference in the design indexes of proportioning, design methods, optimal mixture composition, and water resistance enhancement methods for magnesium chloride cement-stabilized material for road pavement grassroots. Therefore, the research requirements are not yet satisfied for the practical application and theory of road pavements at the grassroots level. In this study, MOC is used as a binding material, and the mixture formed by adding water and crushed stone together is called MOC-stabilised crushed stone material, and we aim to utilize it as the research object and to develop a method of proportion design of MOC-stabilized crushed stone based on the response surface method (RSM) by determining the control indexes of the proportion design of MOC-stabilized crushed stone through the unconfined compressive strength and water resistance tests. We also aim to determine the optimum mixing composition and its water resistance enhancement method, and to verify the applicability and feasibility of its application in the pavement base layer by using ordinary silicate cement-stabilized crushed stone as the control object. This study provides a basis and reference for the application of MOC-stabilized crushed stone material in pavement subgrades.

## Materials and methods

### Raw materials

#### Crushed stone

**Figure 1:**
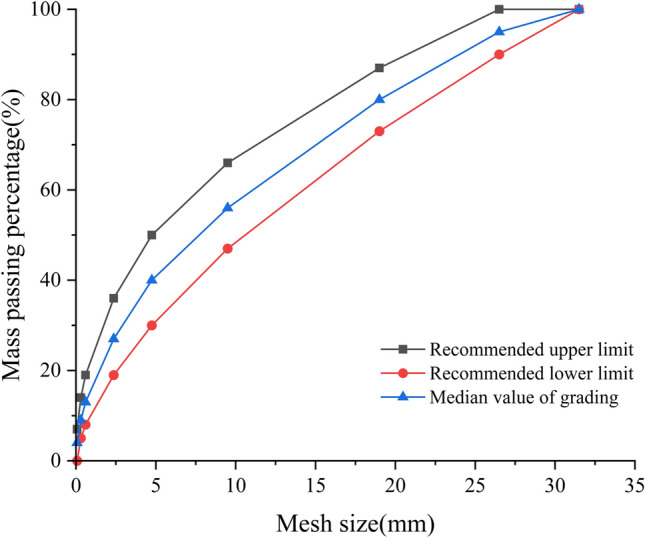
Gradation of crushed stone particles.

Limestone is used as the crushed stone, which is produced in a quarry in Changchun City. The particle size distribution type is the C–C-2 median value recommended by the "Technical Guidelines for Construction of Highway Roadbases" (JTG/T F20-2015). Figure 1 depicts the gradation curve.

#### Active magnesium oxide

**Figure 2:**
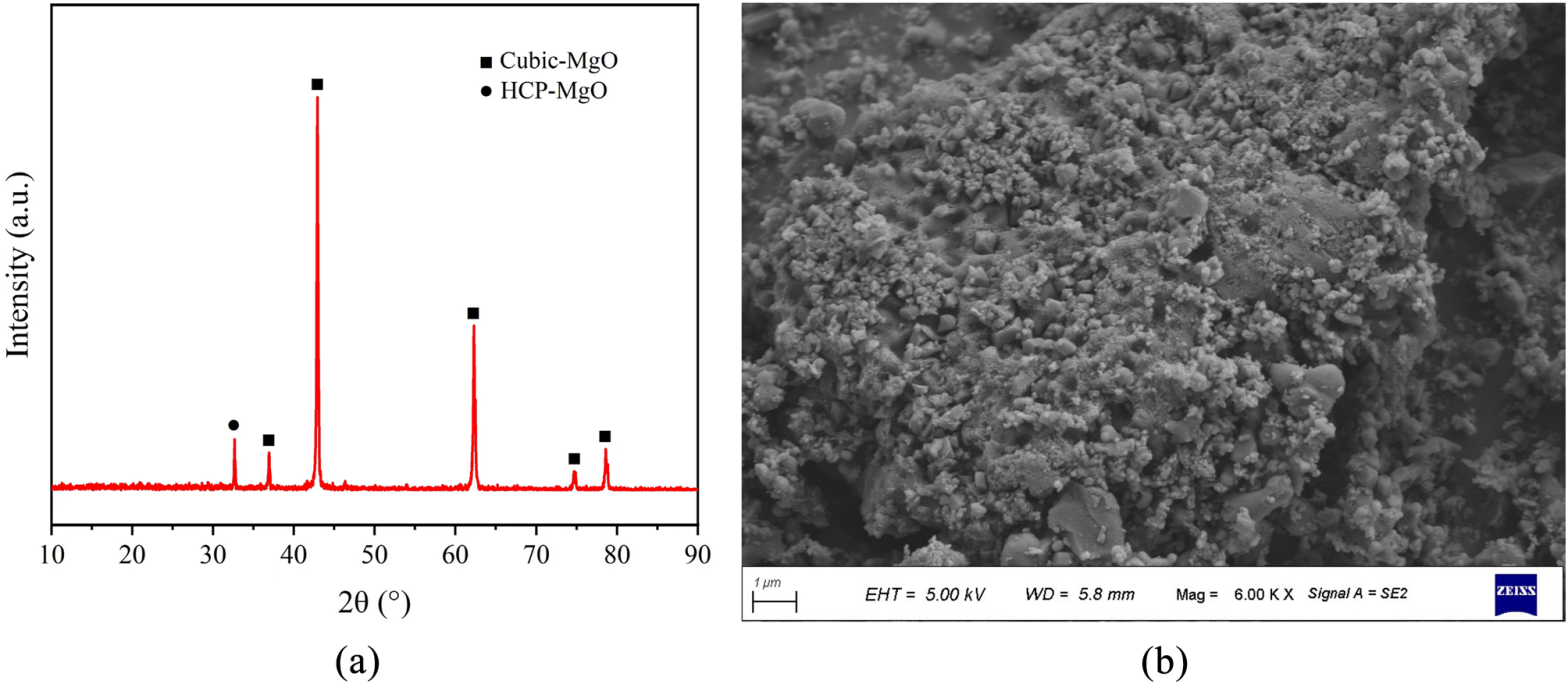
MgO XRD analysis and SEM scans. (**a**) XRD analysis of MgO, (**b**) SEM image of MgO.

MOC is formed by mixing active magnesium oxide powder and a magnesium chloride solution in a certain ratio. We used 98% pure light-burned magnesium oxide powder produced by Dayiqiao City Tianyi Refractory Materials Co., Ltd.; its activity was 63.1%, as detected by the hydration method. Table 1 lists the main components. Figure 2 depicts the XRD pattern and SEM scan of the active MgO.

**Table 1:** Chemical composition of MgO.

MgO/%	SiO_2_/%	CaO/%	lgI/%
≥ 98	≤ 0.4	≤ 0.2	≤ 0.6

#### Magnesium chloride hexahydrate

**Figure 3:**
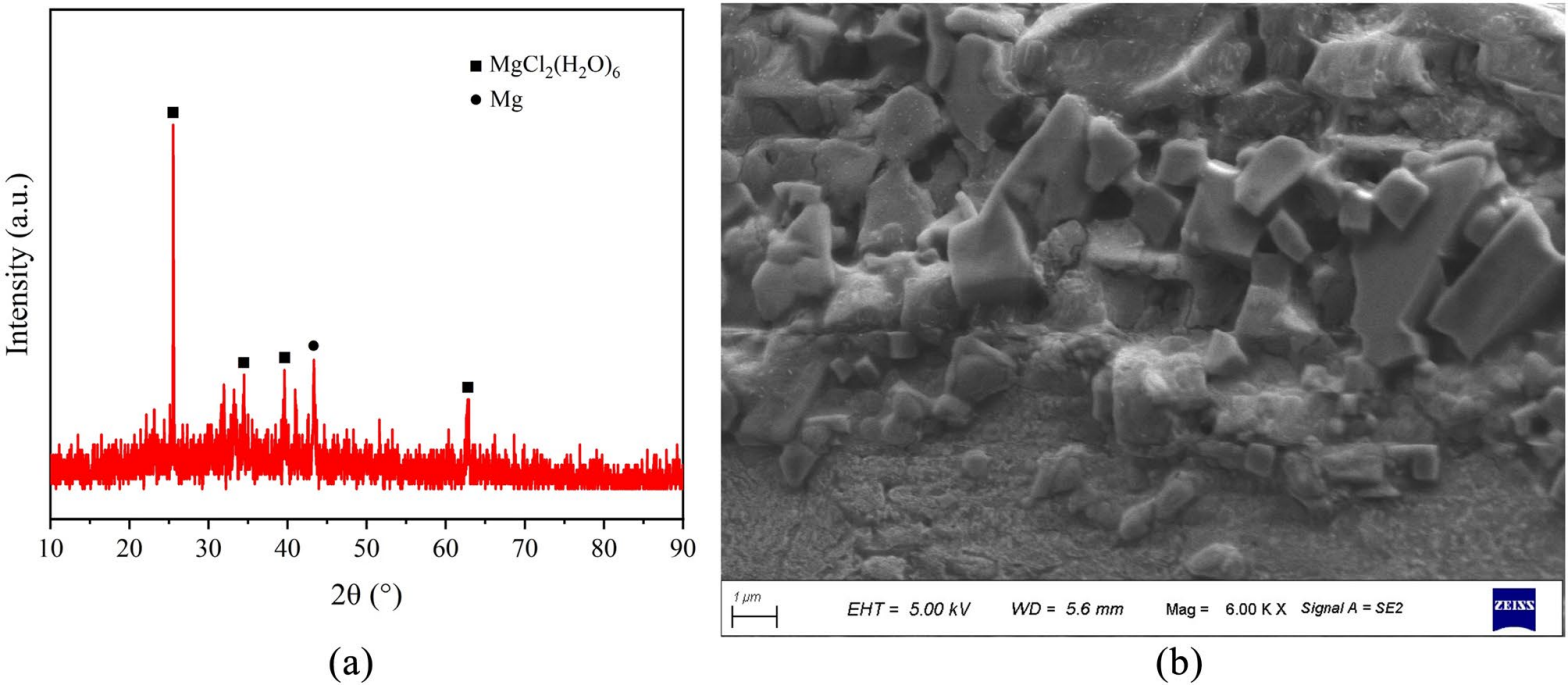
MgCl_2_∙6H_2_O XRD analysis and SEM scans. (**a**) XRD analysis of MgCl_2_∙6H_2_O, (**b**) SEM image of MgCl_2_∙6H_2_O.

Magnesium chloride hexahydrate is an industrial-grade magnesium chloride product produced by Dayiqiao City Tianyi Refractory Materials Co., Ltd. with a purity of 99.8% and an effective magnesium chloride content of 45.68%. Table 2 lists the primary components and Fig. 3 depicts the XRD analysis and SEM images of magnesium chloride hexahydrate.

**Table 2:** Chemical composition of MgCl_2_∙6H_2_O.

MgCl_2_/%	KCl/%	NaCl/%	CaCl_2_/%	MgSO_4_/%
45.68	≤ 0.70	≤ 1.50	≤ 1.00	≤ 2.00

#### Modifiers

We used six modifiers in the experiment: fly ash, citric acid, silica fume, phosphoric acid, waterborne polyurethane, and potassium dihydrogen phosphate. The silica fume used is 96% pure high-activity silica fume produced by Henan Yixiang New Materials Co., Ltd., the citric acid used is 98% pure citric acid produced by Xinqing Technology Co., Ltd., the fly ash used is Grade I fly ash produced by Gongyi City Borun Refractory Materials Co., Ltd., the phosphoric acid used is industrial grade 85% pure phosphoric acid produced by Shandong Junguan Chemical Co., Ltd., the acidic phosphate used is potassium dihydrogen phosphate produced by Tianjin City Dengfeng Chemical Reagent Factory, and the polyurethane used is water-based polyurethane produced by Yoshida Chemical.

#### Portland cement

We used M32.5 Portland cement produced by Changchun Yatai Cement Liability Co., Ltd. Table 3 lists the main technical indicators.

**Table 3:** Main technical indicators of cement.

Initial setting time/min	Final setting time/min	Fineness	Stability
160	300	4.2	qualified

### Test scheme

**Figure 4:**
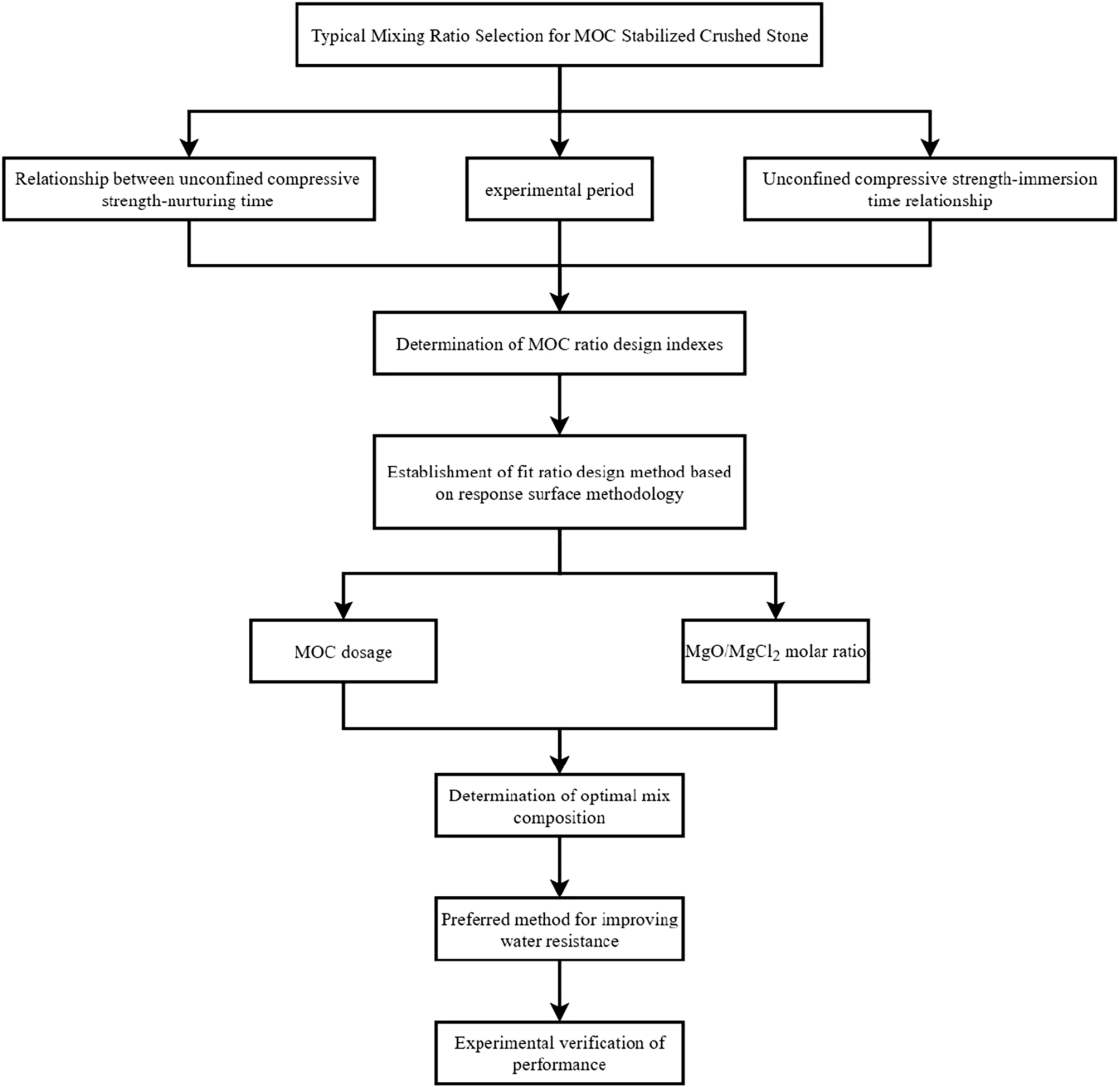
Flow chart of test method.

Considering the characteristics of MOC-stabilized materials and the actual use requirements of road subgrades, the experimental research objective of this study is as follows. Firstly, the typical mixtures were selected, test samples were formed, and unconfined compressive strength and water resistance tests were conducted based on the exploratory experimental results. The control indicators of the composition design of the MOC-stabilized crushed stone mixtures were determined via the analysis of the correlation between the unconfined compressive strength, curing time, and immersion time. A two-factor three-level experiment was then conducted to optimize the mix ratio of MOC-stabilized crushed stone by using the response surface method design with the MOC content and the MgO/MgCl_2_ molar ratio as the influencing factors and the unconfined compressive strength and water resistance coefficient as the response values. We considered fly ash, citric acid + silica fume, phosphoric acid + water-based polyurethane, and potassium dihydrogen phosphate as the four modification schemes. Subsequently, we compared and analysed the water resistance improvement effects and their impact on the strength under different mix ratios of each modification scheme and comprehensively analysed them to determine the best modification method and mixing ratio for improving the water resistance of MOC-stabilized crushed stone. Lastly, the unconfined compressive strength and water resistance coefficient were compared and analysed to verify the applicability and feasibility of MOC-stabilized crushed stone in road subgrades using ordinary Portland cement-stabilized crushed stone with a cement dosage of 5% as a reference. Figure 4 presents a flowchart of the test method.

### Test methods

#### Preparation of test samples

The test specimen was cylindrical, with a diameter and height of 150 mm each. We considered the compaction standard of grassroots materials in the Technical Rules for Highway Pavement Grassroots Construction (JTG/T F20-2015) and the actual control of water content in the construction of pavement grassroots level. Consequently, we conducted a heavy compaction test to measure the maximum dry density of magnesium chloride cement-stabilized crushed stone and the optimal water content at different mixing ratios, and to calculate the mass of crushed stone required for each mixing ratio, along with the amount of active magnesium oxide and magnesium chloride hexahydrate. Based on these results of compaction tests for each proportion of MgO cement-stabilized crushed stone (Table 4), the test specimens were molded using the static compaction method corresponding to 98% compaction and optimal moisture content, and then demolded after two hours of resting. Subsequently, the surface integrity of the specimen was observed and the specimen quality and height were measured, and the specimens that met the requirements were sealed with plastic film. These specimens were then placed in the standard incubator to maintain constant temperature and humidity after being marked and numbered.

**Table 4:** Compaction test results.

Number	MOC /%	MgO/ MgCl_2_	Optimum moisture content /%	Maximum dry density /(g·cm^-3^)
C1	2	5	4.8	2.359
C2	2	6	4.8	2.355
C3	2	7	4.8	2.350
C4	2	7.5	4.7	2.347
C5	2	9	4.7	2.344
C6	4	5	5.2	2.368
C7	4	6	5.1	2.357
C8	4	7	5.1	2.353
C9	4	7.5	5.1	2.350
C10	4	9	5.0	2.346
C11	6	5	5.6	2.388
C12	6	6	5.6	2.380
C13	6	7	5.5	2.373
C14	6	7.5	5.5	2.370
C15	6	9	5.4	2.365
C16	8	5	6.1	2.424
C17	8	6	6.0	2.419
C18	8	7	5.9	2.406
C19	8	7.5	5.9	2.403
C20	8	9	5.8	2.392

#### Unconfined compressive strength test

An unconfined compressive strength test is conducted using a road material strength tester at a loading rate of 1 mm/min. The specific test process is as follows. The test sample that reaches the predetermined curing age is removed, along with the plastic film on the surface; it is immersed in water for 24 h, removed it, absorb the water on the sample surface with a soft cloth, place it on the road-material strength tester, and conduct the loading test until the specimen breaks. The peak pressure of each sample was recorded and its unconfined compressive strength was calculated.

#### Water resistance test

Two sets of test samples were simultaneously formed under the same test conditions. The height and weight were measured after reaching a predetermined constant temperature and humidity curing age. One set was placed directly on the road material strength tester for unconfined compressive strength testing. The other set is immersed in water at 20 ℃ ± 2 ℃, while ensuring that the water level is approximately 2.5 cm above the sample surface. The test samples were removed after reaching a predetermined immersion time, the water on the sample surface was absorbed with a soft cloth, and an unconfined compressive strength test was conducted. The ratio of the unconfined compressive strength under the immersion condition at the same constant temperature and humidity curing age to the unconfined compressive strength under the non-immersion condition was used as the water resistance coefficient. This is used as a technical indicator to evaluate the water resistance of MOC-stabilized crushed stone materials [Eq. (1)].

1 $${\text{K}}_{\text{c}}\text{=}\frac{{\text{R}}_{\text{w}}}{{\text{R}}_{\text{d}}}$$

${\text{K}}_{\text{c}}$ denotes the water resistance coefficient, ${\text{R}}_{\text{w}}$ denotes the unconfined compressive strength under immersion conditions (in MPa), and ${\text{R}}_{\text{d}}$ denotes the unconfined compressive strength under non-immersion conditions (in MPa).

## Design of mixed material composition based on response surface methodology

### Response surface methodology

Response surface methodology (RSM) is a test design method that is suitable for addressing nonlinear data processing-related issues by integrating the advantages of orthogonal experiments and regression analysis. It involves approximating the construction of a polynomial with a clear expression to represent the implicit functional relationships. This method can fit a complex unknown functional relationship in a certain region using a simple linear or quadratic polynomial model, thereby establishing the functional relationship between the factors and response values. Therefore, it is widely used for the material-ratio design optimization^28–30^. RSM is divided into central composite design (CCD) and Behnken design (BBD). The CCD method is implemented to optimize experiments with two or more factors. It presents a higher prediction accuracy despite requiring more experiments with the same number of factors. If the optimal value does not lie within the selected range, the test results can continue to be optimized within the new range, indicating continuity. Conversely, BBD is suitable for optimizing experiments using three or more factors; it requires fewer tests and is more efficient. However, the optimal value must be obtained within the range, and the test results are not continuous.

### Determination of control indicators for mixed material composition design

For the mixed-material design of inorganic binder-stabilized materials, the "Technical Guidelines for Construction of Highway Roadbases" (JTG/T F20-2015) stipulates a 7D unconfined compressive strength as the control indicator. However, existing studies have reported that MOC-stabilized materials exhibit characteristics of rapid early strength growth, high proportions, and poor water resistance^31–36^. The suitable material ratio for the actual pavement base requirements may not be accurately determined by directly applying this indicator, and its water resistance cannot be considered. Therefore, we employ both the unconfined compressive strength and water resistance coefficient as the control indicators for the design of an MOC-stabilized crushed stone mix, considering the strength formation characteristics of MOC-stabilized materials and the water stability requirements of pavement base materials. We aimed to determine the curing age of the unconfined compressive strength and soaking time of the water resistance coefficient as the design control indicators by analysing the relationship between the strength, curing time, and soaking time.

The reference data on the strength growth rule of MOC-stabilized crushed stone and its reasonable proportion are currently limited. The typical proportions of MOC-stabilized crushed stone are selected to analyse its relationship with the curing time and soaking time, given that the optimal MgO/MgCl_2_ molar ratio of magnesia oxychloride cement is 6–9^1–5^. Furthermore, the main hydration product of magnesium chloride and magnesium oxide is 5 Mg(OH )_2_∙MgCl_2_∙8H_2_O and 3 Mg (OH )_2_∙MgCl_2_∙8H_2_O, which are termed as the 5-phase and 3-phase crystals, respectively, and their chemical reaction formula is given as:

2 $${\text{5MgO}} + {\text{MgCl}}_{{2}} + {\text{13H}}_{{2}} {\text{O}} \to {\text{5Mg }}\left( {{\text{OH}} \cdot } \right)_{{2}} \cdot {\text{MgCl}}_{{2}} \cdot {\text{8H}}_{{2}} {\text{O}}$$

3 $${\text{3MgO}} + {\text{MgCl}}_{{2}} + {\text{11H}}_{{2}} {\text{O}} \to {\text{3Mg }}\left( {{\text{OH}} \cdot } \right)_{{2}} \cdot {\text{MgCl}}_{{2}} \cdot {\text{8H}}_{{2}} {\text{O}}$$

The mechanical strength of the 5-phase crystal is evidently higher than that of the 3-phase crystal, which is the main strength index of the MOC. The 3-phase and 5-phase crystals are both mesostable; the 5-phase crystal can be transformed into the 3-phase crystal, which can eventually be decomposed to Mg(OH)_2_. Additionally, some of the remaining MgO reacted with water to generate water magnesite; its chemical reaction formula is given below:

4 $${\text{MgO}} + {\text{ H}}_{{2}} {\text{O}} \to {\text{Mg }}\left( {{\text{OH}}} \right)_{{2}}$$

An appropriate molar ratio can promote the magnesium chloride and magnesium oxide reactions to generate more 5-phase crystallization and enhance the strength of the magnesium chloride cement. The cement dosage of ordinary Portland cement-stabilized crushed stone in engineering practice is generally 3%-9%. Therefore, in this study, we designed a comprehensive exploratory experiment with MgO/MgCl_2_ molar ratios of 6, 7.5, and 9, and MOC dosages of 4%, 6%, and 8% using the 7D unconfined compressive strength as an indicator.

**Figure 5:**
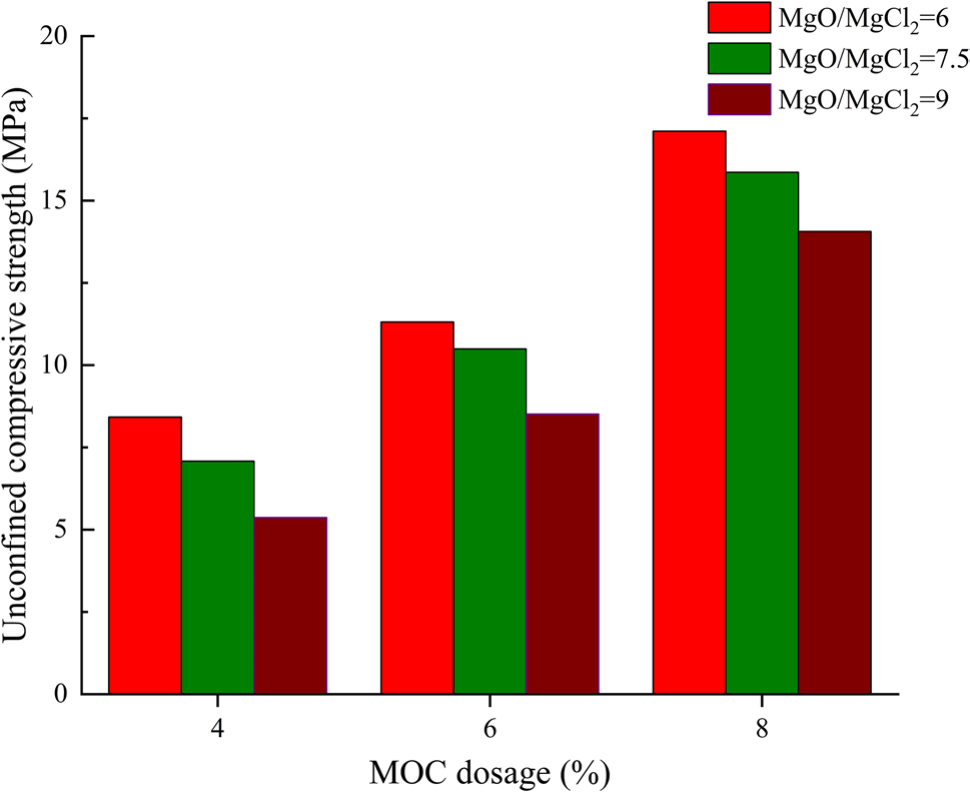
Exploratory test results.

The 7D unconfined compressive strength of the MOC-stabilized crushed stone decreases with an increase in the MgO/MgCl_2_ molar ratio and increases with an increase in the MOC content (Fig. 5). Therefore, we selected MgO/MgCl_2_ molar ratios of 9 + MOC 4%, MgO/MgCl_2_ molar ratios of 7.5 + MOC 6%, and MgO/MgCl_2_ molar ratios of 6 + MOC 8% as the three typical proportioning test samples to reduce the experimental workload and ensure the representativeness of the experimental data. We performed unconfined compressive strength tests and water resistance tests to further analyse the relationship between the strength, curing time, and soaking time of the MOC-stabilized crushed stone. These tests provide data for determining the control indicators required for the composition design of the MOC-stabilized crushed stone mix.

**Figure 6:**
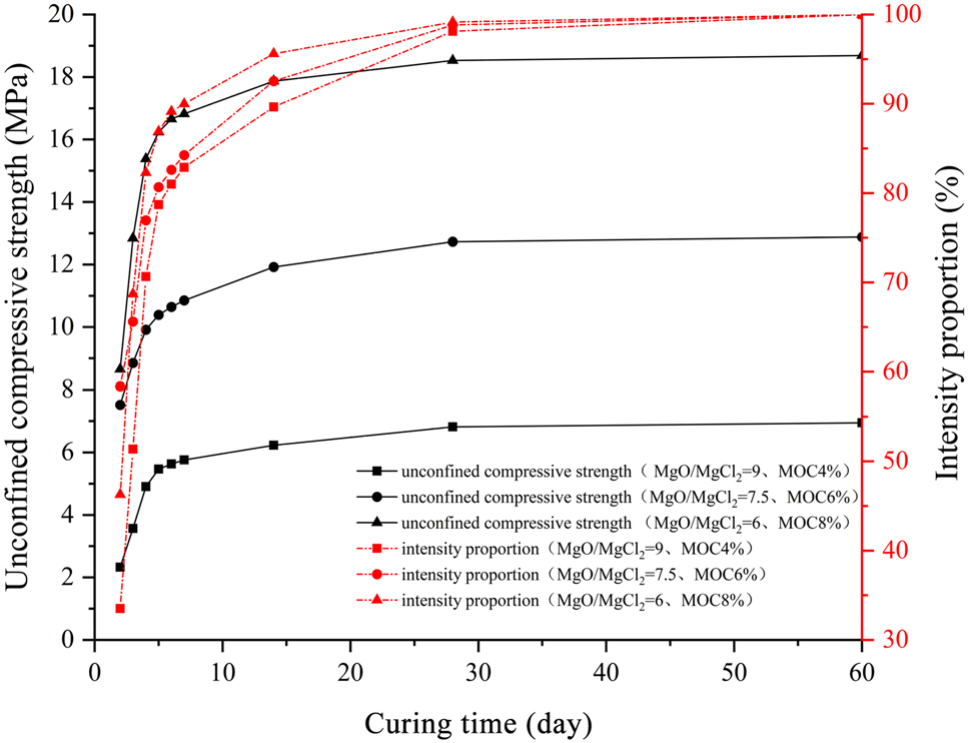
Strength development trend of MOC-stabilized crushed stone.

**Figure 7:**
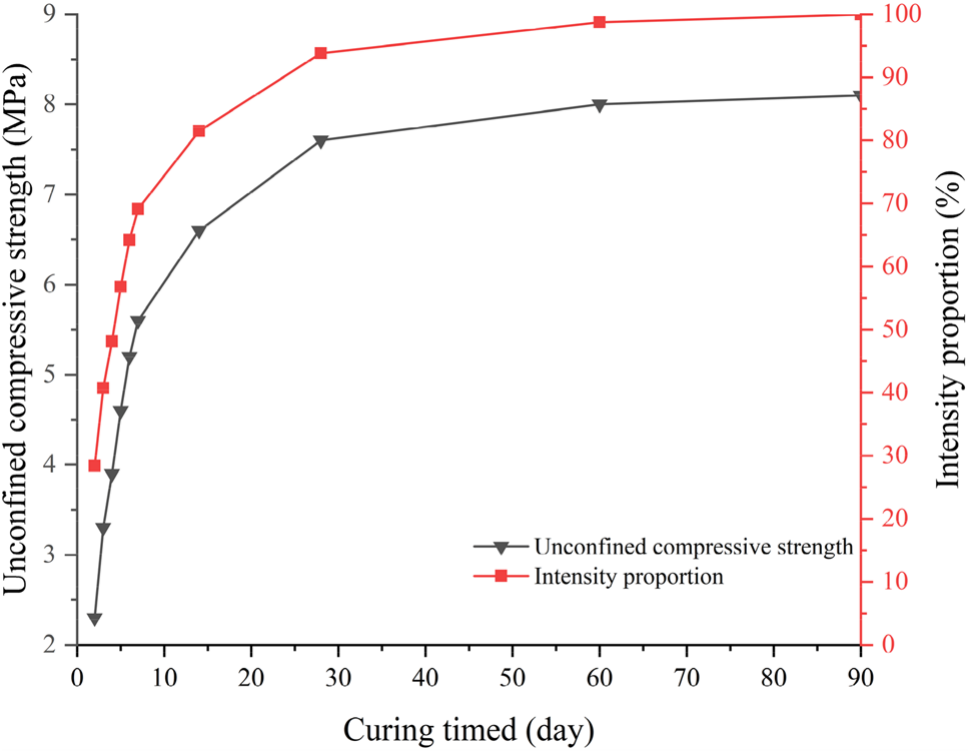
Strength development trend of 5% Portland cement-stabilized crushed stone.

The unconfined compressive strength of the MOC-stabilized crushed stone presents the fastest strength growth rate in the first four days (3 days of constant temperature and humidity curing + 1 day of soaking curing) (Fig. 6). The strength growth over 514 days gradually becomes flat, and the strength growth proportion after 14 days was limited. By 28 days, it reached the final strength, after which there was no significant increase in the strength. This is in line with the findings of Sun and Qiao. that MOC develops rapidly and with high strength in the early stages and grows slowly in the later stages^27,31^. Therefore, the MOC-stabilized crushed stone material must use the 28-day curing age unconfined compressive strength as its final strength. Ordinary Portland cement-stabilized crushed stone presents a faster strength growth rate in the first 7 days, and the strength growth during 8 ~ 28 days gradually becomes flat, with slow growth after 28 days (Fig. 7). For cement-stabilized crushed stone materials, the "Technical Guidelines for Construction of Highway Roadbases" (JTG/T F20-2015) clearly stipulate that the 7-day unconfined compressive strength must be used as a mixed material composition design and construction quality control technical indicator. In engineering practice, the 90-day curing age strength is generally considered as the final strength. From the experimental results, the 7-day unconfined compressive strength of ordinary Portland cement-stabilized crushed stone accounts for 69.1% of its 90-day final strength, while the strength formed within 4 days for the three typical proportions of MOC-stabilized crushed stone materials reached 72.1%, 77.8%, and 82.3% of their 28-day final strengths, all above 70%. Based on this and considering the material strength formation characteristics and experimental cycle issues, using the 4-day unconfined compressive strength ${\text{R}}_{\text{4d}}$ (3 days of constant temperature and humidity curing + 1 day of soaking curing) as the strength control indicator for the composition design of the MOC-stabilized crushed stone mix is advisable.

**Figure 8:**
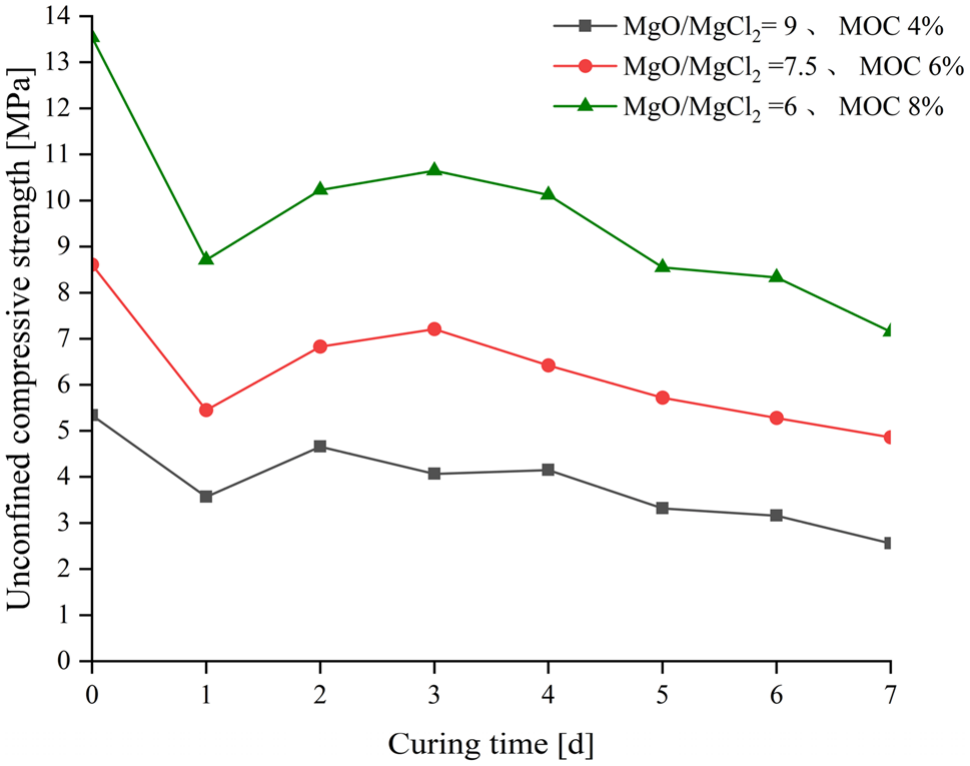
Change in unconfined compressive strength of MOC-stabilized crushed stone with soaking time.

Based on the previously mentioned study conducted on the change in the unconfined compressive strength of MOC-stabilized crushed stone as a function of the curing time, water resistance tests were conducted on the test specimens that were cured at constant temperature and humidity for 3 days to determine the specific soaking time for the water resistance coefficient. Their unconfined compressive strength was tested after no soaking, 1 day of soaking, and 2 ~ 7 days of soaking, respectively. The results (Fig. 8) indicate that the unconfined compressive strength of the MOC-stabilized crushed stone material decreased significantly after 1 day of soaking. When compared with the strength without soaking, the strengths of the three typical mixture proportions decreased by 33.1%, 37.3%, and 36.3%, respectively. The strength increased slightly after 2–3 days of soaking but generally decreased with the increase in the soaking time. Therefore, considering factors such as the age of strength control, pattern of change in the unconfined compressive strength with soaking time, and test cycle, the appropriate index for evaluating the water resistance performance of MOC-stabilized crushed stone must be based on 3 days of constant temperature and humidity curing and a 1-day soaking test condition. It is denoted as ${\text{K}}_{\text{c1d}}$.

The selection of 4-day unconfined compressive strength and the 1-day water resistance coefficient as the design control index of MgO cement-stabilized crushed stone considerably reduces the period required for subsequent tests. It also conforms to the characteristics of magnesium chloride cement, which presents a fast growth rate of strength in the early stage and a weak growth rate of strength in the later stage. This ensures that the final strength of the magnesium chloride cement-stabilized crushed stone can satisfy the expected design requirements in actual engineering applications as a pavement subgrade material. It also helps in the evaluation and consideration of the water resistance performance of magnesium chloride cement-stabilized crushed stone. Furthermore, the water resistance of the MgO cement-stabilized crushed stone was evaluated and considered, which can better verify its strength stability when applied to a wet environment that the pavement base material may face.

### Design of mixture composition based on response surface methodology

#### Design of experiment scheme using response surface methodology

Considering the characteristics of the CCD and BBD, along with the main factors involved in the design being the MgO/MgCl_2_ ratio and the MOC-molar ratio, the composition design of MOC-stabilized crushed stone was optimized using CCD. The 4-day unconfined compressive strength, ${\text{R}}_{\text{4d}}$, and the 1-day water resistance coefficient, ${\text{K}}_{\text{c1d}}$, were used as the response indicators, and the MgO/MgCl_2_ and MOC molar ratios were considered as the influencing factors for the two-factor, three-level, two-response experimental design. Table 5 lists the levels of the influencing factors of the MgO/MgCl_2_ molar ratio and MOC dosage based on the strength test results of the three typical mixtures of MOC-stabilized crushed stone and the actual strength requirements of the pavement base materials. To minimize the impact of errors, five centre points and eight boundary points were set for 13 sets of test specimens. Each set contained 18 test samples, of which nine were used to test and determine the unconfined compressive strength, ${\text{R}}_{\text{4d}}$, and nine were used to test the unconfined compressive strength under the non-soaked conditions to calculate and determine the water resistance coefficient, ${\text{K}}_{\text{c1d}}$. The average value of nine sample test results was used as the response indicator value (Table 6).

**Table 5:** Levels of influencing factors.

Influence factor	Number	Level		
		-1	0	1
MgO/MgCl_2_	A	5	6	7
MOC/%	B	2	4	6

**Table 6:** Experimental scheme and summary of results.

number	A	B/%	${\text{R}}_{\text{4d}}$/MPa	${\text{K}}_{\text{c1d}}$
1	7	4	6.02	0.69
2	7	6	8.86	0.71
3	6	4	6.96	0.80
4	6	4	6.71	0.82
5	5	6	8.43	0.73
6	6	4	7.33	0.78
7	6	4	6.99	0.81
8	5	4	5.29	0.75
9	6	4	6.52	0.79
10	6	2	2.63	0.75
11	6	6	9.38	0.76
12	5	2	2.19	0.72
13	7	2	2.11	0.68

#### Regression equation establishment and analysis of variance

Following a preliminary analysis of the results, we proposed a second-degree polynomial function of the two variables to characterize the relationship between the unconfined compressive strength and the water resistance coefficient response indicators and their influencing factors, as shown in Eq. (5). The least-squares method was employed for the nonlinear regression fitting to construct a prediction model for the unconfined compressive strength and water resistance coefficient of the MOC-stabilized crushed stone.

5 $$Y={\beta }_{0}+\sum {\beta }_{i}{X}_{i}+\sum {\beta }_{ii}{X}_{i}^{2}+\sum {\beta }_{ij}{X}_{i}{X}_{j}$$

Y denotes the response indicator, ${\beta }_{0}$ denotes the intercept of the response indicator, ${\beta }_{i}$ denotes the linear coefficient of influencing factor, ${\beta }_{ii}$ denotes the quadratic coefficient of influencing factor, ${\beta }_{ij}$ denotes interaction coefficient of influencing factors.

The effectiveness of the selected predictive model directly determines the quality and level of the mixture composition design based on the response surface method. Variance analysis and significance tests were conducted on each item in the selected model to verify the rationality and applicability of the aforementioned model and to exclude insignificant items during the data fitting to simplify the prediction model (Tables 7 and 8).

**Table 7:** Analysis of variance for unconfined compressive strength regression model.

Source	Sum of squares	Degree of freedom	F-value	P-value
A	0.19	1	17.40	0.0229
B	64.94	1	580.25	< 0.0001
AB	0.065	1	0.58	0.0478
A^2^	2.43	1	21.72	0.0023
B^2^	0.96	1	8.54	0.0233
Residual	0.78	7	—	—
Lack of fit	0.41	3	1.44	0.3567
Pure error	0.38	4	—	—
Sum	71.31	12	—	—

**Table 8:** Analysis of variance for water resistance coefficient regression model.

Source	Sum of squares	Degree of freedom	F-value	P-value
A	2.400 × 10^–3^	1	7.71	0.0274
B	4.167 × 10^–4^	1	1.34	0.2852
AB	1.000 × 10^–4^	1	0.32	0.5885
A^2^	0.011	1	35.34	0.0006
B^2^	2.181 × 10^–3^	1	7.01	0.0331
Residual	2.178 × 10^–3^	7	—	—
Lack of fit	1.178 × 10^–3^	3	1.57	0.3282
Pure error	1.000 × 10^–3^	4	—	—
Sum	0.025	12	—	—

We determined the significance level of the effect of each factor item on the unconfined compressive strength and water resistance coefficient response indicators of the MOC-stabilized crushed stone based on the P-value. We employed a threshold significance level of $\upalpha = 0.05$. When ${\text{P}} \le {0}{\text{.05}}$, the impact of the factor on the response indicator was considered to be significant^37^. Otherwise, it is considered insignificant and can be eliminated during nonlinear regression fitting. The P-values for A, B, AB, A^2^, and B^2^ were all less than 0.05, as shown in Tables 7 and 8, indicating that the ratios of MgO/MgCl_2_, amounts of MOC, their interaction, and quadratic terms all significantly influenced the unconfined compressive strength response indicator. For A, A^2^, and B^2^, the P-values were all < 0.05. However, the P-values for B and AB were greater than 0.05, indicating that the square terms of the MgO/MgCl_2_ ratio and MOC amount significantly affected the water resistance coefficient response indicator, whereas the interaction between the MgO/MgCl_2_ ratio and MOC amount did not. Based on these results, the prediction models for the unconfined compressive strength and water resistance coefficient were constructed as follows:

6 $${\text{R}}_{\text{4d}}\text{=0.18 }{\text{A}}\text{+3.29 }{\text{B}}\text{+0.13 }{\text{AB}}\text{-0.94}{\text{ A}}^{2}\text{- 0.59 }{\text{B}}^{2}\text{+6.81}$$

7 $${\text{K}}_{\text{c1d}}\text{=-0.02 }{\text{A}}\text{-0.063 }{\text{A}}^{2}\text{-0.028 }{\text{B}}^{2}\text{+0.80}$$

The correlation coefficients of the two fitted equations were 0.9812 and 0.9084, indicating that the established predictive models could accurately reflect the relationship between the unconfined compressive strength, water resistance coefficient, MOC amount, and MgO/MgCl_2_ molar ratio. They can be used to determine the optimal mixture composition of the MOC-stabilized crushed stone. Additionally, the signal-to-noise ratios (AP) for the two equations were 33.310 and 11.422, respectively, which were much greater than the required value of 4, further demonstrating the reliability and accuracy of the prediction models.

**Figure 9:**
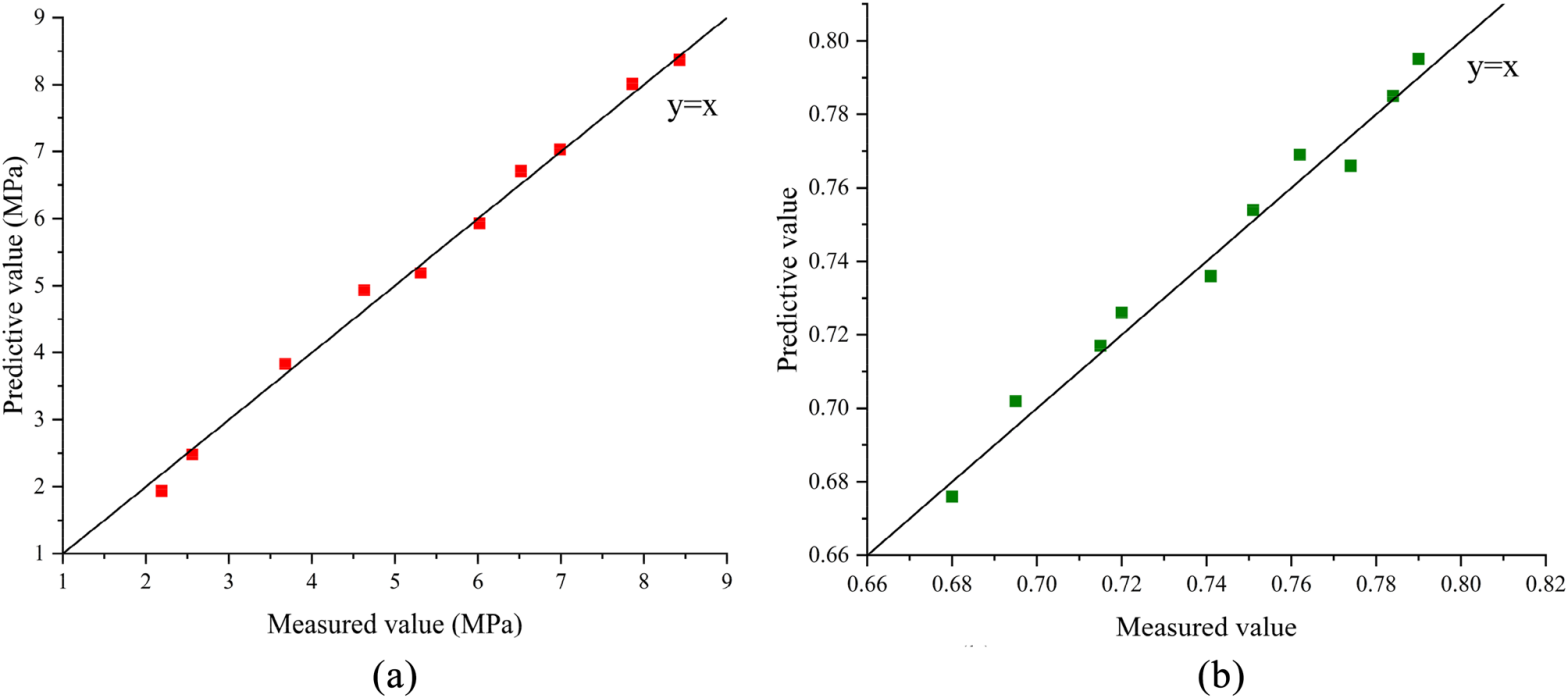
Distribution plot of response indicator-predicted actual values. (**a**) Unconfined compressive strength, (**b**) Water resistance coefficient.

To further verify the reliability and generalization ability of the prediction model, 10 sets of ratio parameters were designed, which varied from the above fitting data. The 4-day unconfined compressive strength and 1-day softening coefficient were measured for the indoor-formed samples, and the predicted values were calculated using Eqs. (3) and (4). The measured and predicted values for each ratio parameter are plotted on the same graph. The data points for the unconfined compressive strength and softening coefficient of the MOC-stabilized crushed stone were located near the line, indicating that their predicted values were close to the measured values (Fig. 9). The established prediction model exhibits high reliability and generalization ability and can be used for the composition design of MOC-stabilized crushed stone mixtures.

#### Response model analysis

**Figure 10:**
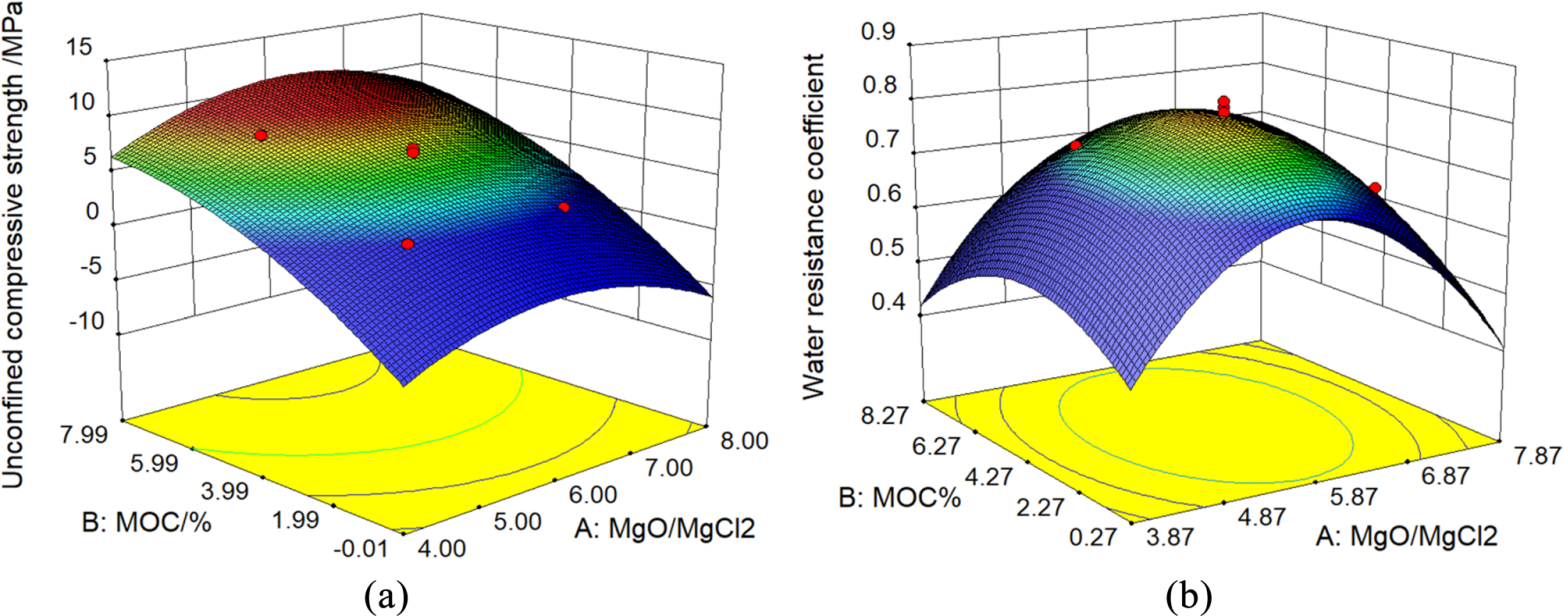
Response surface models. (**a**) 4d Unconfined Compressive Strength, (**b**) Water Resistance Coefficient.

The RSM were established for the 4-day unconfined compressive strength and 1-day softening coefficient of MOC-stabilized crushed stone using the CCD method under the interactive effects of the MOC amount and MgO/MgCl_2_ molar ratio (Fig. 10). The 4-day unconfined compressive strength of MOC-stabilized crushed stone increased continuously with an increase in the MOC amount (Fig. 10a). As the MgO/MgCl_2_ molar ratio increased, there was a trend of first increasing and then decreasing, indicating an optimal MgO/MgCl_2_ molar ratio at a certain MOC amount. Additionally, the influence of the amount of MOC on the unconfined compressive strength is much greater than that of the MgO/MgCl_2_ molar ratio, which concurs with the aforementioned variance analysis results. Figure 10b shows that the water resistance coefficient initially increased and then decreased with an increase in both the MgO/MgCl_2_ molar ratio and the MOC amount. The maximum value on the response surface corresponded to the optimal MgO/MgCl_2_ molar ratio and MOC amount. Furthermore, the MgO/MgCl_2_ molar ratio has a more significant effect on the water resistance coefficient than the MOC content.

#### Determination and verification of the optimal mixture composition

Based on the previously established regression equations for the unconfined compressive strength, ${\text{R}}_{{4}{\text{d}}}$, and water resistance coefficient, ${\text{K}}_{\text{c1d}}$, using the point prediction functionality of the response surface method, an optimal mixture composition for the MOC-stabilized crushed stone was determined through the prediction of different response indicators within the range of influencing factors. Considering the actual requirements of the road surface base and the characteristics of materials with high strength and poor water resistance the expected values of the unconfined compressive strength and water resistance coefficient while determining the optimal mixture composition for the MOC-stabilized crushed stone were set as the maximum values of the target function. The importance levels of the unconfined compressive strength and water resistance coefficient were both set to five. Based on the influencing factor-level design in the aforementioned response surface method experimental plan, the range of the molar ratio of MgO/MgCl_2_ was set from 5 to 7, and the range of the MOC content was set from 3 to 5%. Table 9 presents the optimal mixture composition for MOC-stabilized crushed stone determined by the response surface method based on these criteria. Table 10 lists the predicted values of the unconfined compressive strength, ${\text{R}}_{\text{4d}}$, and water-resistance coefficient, ${\text{K}}_{\text{c1d}}$, under the optimal mixture composition.

**Table 9:** Optimal mixture composition of MOC-stabilized crushed stone.

Influence factor	MgO/MgCl_2_ (molar ratio)	MOC dosage/%
Optimal mix composition	5.85	4.27

**Table 10:** Predicted design indicators under the optimal mixture composition.

Design indicators	${\text{R}}_{{4}{\text{d}}}$/MPa	${\text{K}}_{\text{c1d}}$
Predictive value	7.19	0.78

The indoor moulded test samples were tested under equivalent design parameter conditions based on the determined optimal mixture composition to determine the feasibility of the RSM in designing the mixture composition of the MOC-stabilized crushed stone and the reliability of the optimal mixture composition design results (Table 11). A comparative analysis of Tables 10 and 11 demonstrates that the predicted and actual values of the unconfined compressive strength,${\text{R}}_{\text{4d}}$, and water resistance coefficient, ${\text{K}}_{\text{c1d}}$, are fairly consistent under the optimal mixture composition determined by the RSM, with errors limited within 2%. This demonstrates the feasibility of the design of the mixture composition for the MOC-stabilized crushed stone using the RSM, and the selected optimal mixture composition accurately reflects the actual situation of the material.

**Table 11:** Measured values of design indicators under the optimal mixture composition.

Design indicators	${\text{R}}_{{4}{\text{d}}}$/MPa	${\text{K}}_{\text{c1d}}$
Verification value	7.20	0.77

The RSM was used to determine the functional relationship between the unconfined compressive strength and water resistance coefficient of the MOC, and the MOC dosage and MgO/MgCl_2_ molar ratio of the MOC-stabilized crushed stone. A response model was then established, which indicated the intrinsic connection between the influencing factors and the control indexes. The magnitude of the interaction between the factors was analysed, and the optimal mixing proportion of MOC-stabilized crushed stone was determined, which demonstrates the superiority of the RSM in the design and optimization of the mixing proportions under multi-factor and multi-level conditions. The design optimization and validation were performed for the characteristics of magnesium chloroxylate cement, such as rapid strength growth and poor water resistance. This study provides a scientific method to design the mixing composition of the MOC-stabilized crushed stone, a new type of pavement base material, and presents a strong basis for the optimal mixing composition. It also demonstrates the effectiveness and generalizability of the RSM in customizing the material characteristics corresponding to the specific engineering requirements, which presents a method for the design of multifactor and multilevel composite material mixing ratios.

## Water resistance enhancement method

### Experimental plan design

Although MOC-based materials presents advantages such as low carbon emissions, low energy consumption, and high strength, they also have drawbacks such as poor water resistance, making them unsuitable for use in damp environments. Therefore, a practical and effective method must be developed for improving the water resistance of MOC-stabilized crushed stone on road surface bases to enhance its feasibility and application scope. Consequently, we integrate the existing research results on MOC modification methods with the actual requirements of a road surface base. Four modification schemes were adopted based on the previously determined optimal mixture compositions: fly ash, citric acid + silica fume, phosphoric acid + waterborne polyurethane, and dipotassium phosphate salt. By replacing the MOC admixtures on an equal-mass basis, we analysed the changing rules of the unconfined compressive strength, ${\text{R}}_{\text{4d}}$, and water resistance coefficient, ${\text{K}}_{\text{c1d}}$, under different mixing ratios. We aim to determine the optimal additive amounts for each scheme and determine the best modification method for enhancing the water resistance of MOC-stabilized crushed stone. This will ultimately provide a suitable mixture composition for MOC-stabilized crushed stone used in road surface bases. Table 12 presents the experimental research plan.

**Table 12:** Types and amounts of modifiers.

Types of modifiers	Dosage/%				
Fly ash (FA)	5	10	15	20	25
Phosphoric acid + waterborne polyurethane (H3P + WP)	0.5 + 5	1 + 10	1.5 + 15	2 + 20	2.5 + 25
Citric acid + silica fume (CA + SA)	0.5 + 5	1 + 10	1.5 + 15	2 + 20	2.5 + 25
KH_2_PO_4_ (KH2P)	5	10	15	20	25

### Experimental results and analysis

**Figure 11:**
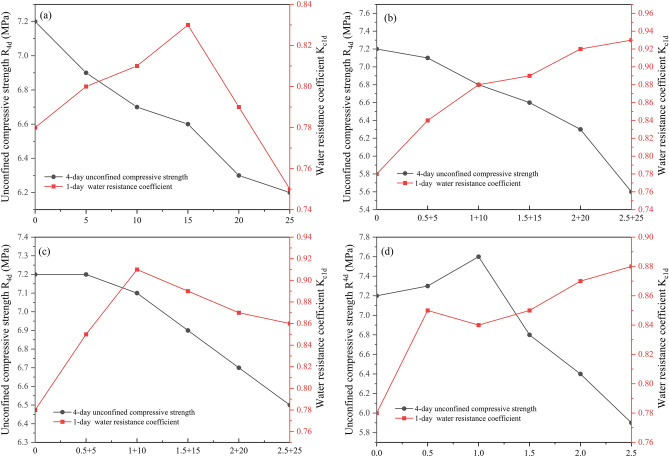
Modification effects of different schemes. (**a**) FA dosage (%), (**b**) H3P+WP dosage (%), (**c**) CA+SA dosage (%), (**d**) KH2P dosage (%).

Figure 11 presents the unconfined compressive strength, ${\text{R}}_{\text{4d}}$, and water resistance coefficient, ${\text{K}}_{\text{c1d}}$, of the MOC-stabilized crushed stone based on the modifier dosage under the four modification schemes.

The unconfined compressive strength of the MOC-stabilized crushed stone decreases gradually with an increase in the fly ash dosage (Fig. 11a). This is because equal mass substitution is used for adding the modifier in this study, wherein the MOC admixture of the optimal mixed material is replaced with the same mass of the modifier. The addition of fly ash reduced the dosage of magnesia oxychloride cement. However, fly ash reacts slowly in an alkaline activation environment, causing a decline in the early strength. The water resistance coefficient first increases and then decreases with the increase in the fly ash content, peaking at 15%. This is because fly ash is an active material with a microaggregate effect. This can improve the weak interface between the coarse crushed stone and MOC binders. Additionally, the fly ash filled the gaps between the crushed stones, enhancing their compactness. The dense microstructure of the MOC was primarily attributed to the filling effect of fine fly ash particles and the formation of Mg-Cl-Si–H gel. This increases the water resistance of the MOC-stabilized crushed stone to a certain extent^8,11,13,36,38^. However, when the fly ash content surpasses a certain proportion, the excessive fly ash increases the proportion of harmful pores, adversely affecting the water resistance.

The water resistance of the MCO-stabilized crushed stone progressively improved, while the strength continuously decreased with an increase in the phosphoric acid and waterborne amino resin contents (Fig. 11b). The improved water resistance is primarily attributed to the coordination reaction between the phosphate ions produced by the hydrolysis of phosphoric acid and Mg^+^. This reduces the minimum concentration requirement of Mg^+^ for the formation of the 5-phase crystal, hinders the formation of Mg(OH)_2_, and reduces the rate of conversion from the 5-phase crystal to the 3-phase crystal. This enhances the stability of the 5-phase crystal, thereby improving the water resistance of the MOC-stabilized crushed stone^14,18,39,40^. Furthermore, waterborne polyurethane, a common waterproofing paint used in construction, exhibits a strong hydrophobicity^21,22,41,42^. When added to magnesia oxychloride cement-stabilized crushed stone, it forms a waterproof film on its surface and interior, effectively improving its water resistance. The continuous decrease in strength was primarily caused by the addition of phosphoric acid and waterborne amino resin, which reduced the proportion of MOC, leading to a reduction in the hydration products.

With an increase in the citric acid and silica fume contents, the strength of the magnesia oxychloride cement-stabilized crushed stone gradually decreases (Fig. 11c), the reason for which is similar to the second modification scheme. The water resistance coefficient initially increased and then decreased with the increase in the modifier dosage, peaking at a dosage of 1% citric acid and 10% silica fume. This is because citrate ions react with the active MgO particle surface [Mg(OH)(H_2_O)_n_]^+^ to form an organic magnesium complex. This reduces the formation rate of Mg(OH)_2_ during the hydration of MgO cement and induces more MgO to participate in the hydration reaction, forming a 5-phase crystal^15,17,43^. The silica fumes, which are characterized by their small particle size and large specific surface area, also exhibit a microaggregate effect. They can uniformly fill the internal pores of the material, enhancing the compactness of MOC-stabilized crushed stone^9,10,12,30^, collectively improving its water resistance. However, when the content of citric acid and silica fume surpasses a certain ratio, the excessive citric acid hinders the hydration process of MOC, thus affecting the normal development of the 5-phase crystal, which affects the growth of the strength of the mixture, and the excessive silica fume affects the formation of the framework structure of the material, adversely affecting its water resistance.

The water resistance coefficient of the MOC-stabilized crushed stone typically exhibits an upward trend with an increase in the potassium dihydrogen phosphate content, while the strength first increased and then decreased (Fig. 11d). The reason for this is similar to the action mechanism of phosphoric acid, wherein the produced phosphate ions coordinate with Mg^+^, thereby reducing the formation of Mg(OH)_2_ precipitates, which is conducive to the formation of the 5-phase crystal, thus enhancing the water resistance of the MOC-stabilized crushed stone. However, exceeding a certain mixing ratio causes a decrease in the early strength of the MOC-stabilized crushed stone.

### Selection of methods for enhancing water resistance

**Figure 12:**
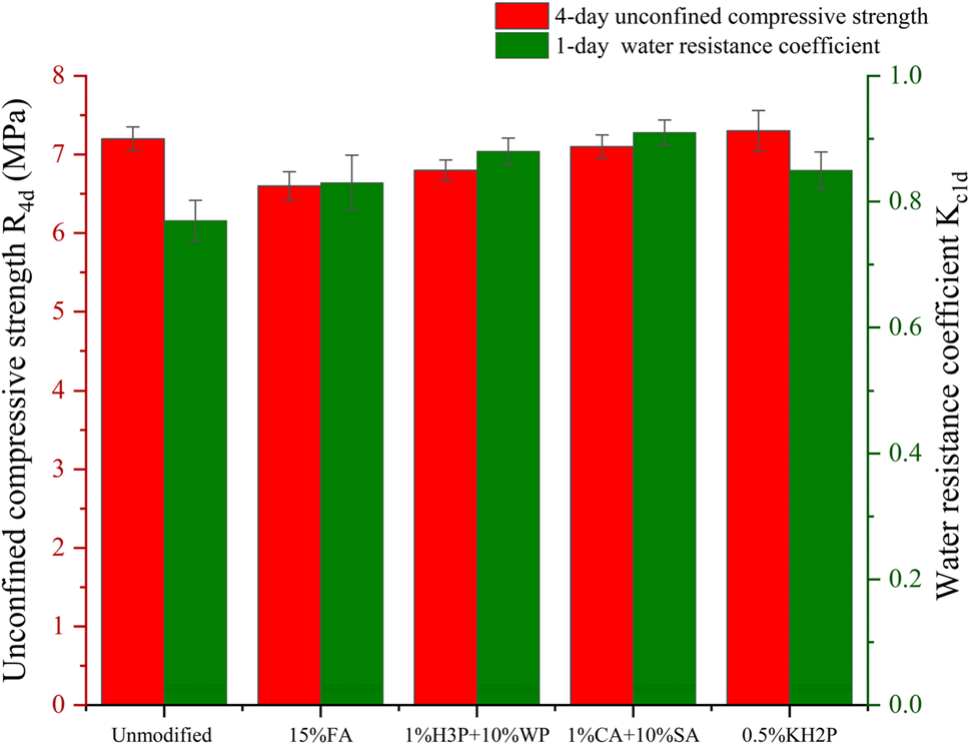
Comparison of effects of different modification methods.

Factors such as improved water resistance performance, strength, and economic feasibility were considered for four modification schemes for MOC-stabilized crushed stone. The optimal amounts of the modifiers for each scheme were 15% (fly ash), 1% + 10% (citric acid + silica fume), 1% + 10% (phosphoric acid + water-based polyurethane), and 0.5% (dihydrogen potassium phosphate). Figure 12 depicts the 4-day unconfined compressive strength, ${\text{R}}_{\text{4d}}$, and the 1-day water immersion water resistance coefficient, ${\text{K}}_{\text{c1d}}$, of the stabilized crushed stone with optimal modifier amounts and without any modification.

Under a reasonable dosage of modifiers in the four schemes, apart from a slight increase in the unconfined compressive strength of 0.5% potassium dihydrogen phosphate, the addition of the other three modifiers adversely affected the strength of the MgO cement-stabilized crushed stone, among which citric acid + silica fume presented the least improvement effect. 1% citric acid + 10% silica fume increased the water resistance coefficient of the MgO cement-stabilized crushed stone from 0.78 to 0.91 before modification to 16.67%, and the improvement effect was the most obvious, while the unconfined compressive strength was only reduced by 0.10 MPa. This satisfies both the water resistance and strength requirements of pavement at the grassroots level, while presenting the advantages of low cost and easy availability of citric acid and silica fume. Thus, we adopted the modification scheme of 1% citric acid + 10% silica fume as the water resistance enhancement method for magnesium chlorooxygenated cement-stabilized stone. However, this review modification method must ensure that small amounts of citric acid and silica fume can be uniformly distributed in the MOC-stabilized crushed stone, which significantly increases the construction difficulty of the road base layer and improves the construction quality.

## Performance test verification

To further verify the feasibility of using the MOC-stabilized crushed stone as a road base and the effectiveness of its water-resistance enhancement method, samples were prepared based on the previously determined optimal mixing composition. The unconfined compressive strength and water resistance tests were conducted after curing for 28 days. These results were compared with those of ordinary Portland cement-stabilized crushed stone (5% cement content, M32.5 cement grade) which was cured for 90 days.

**Figure 13:**
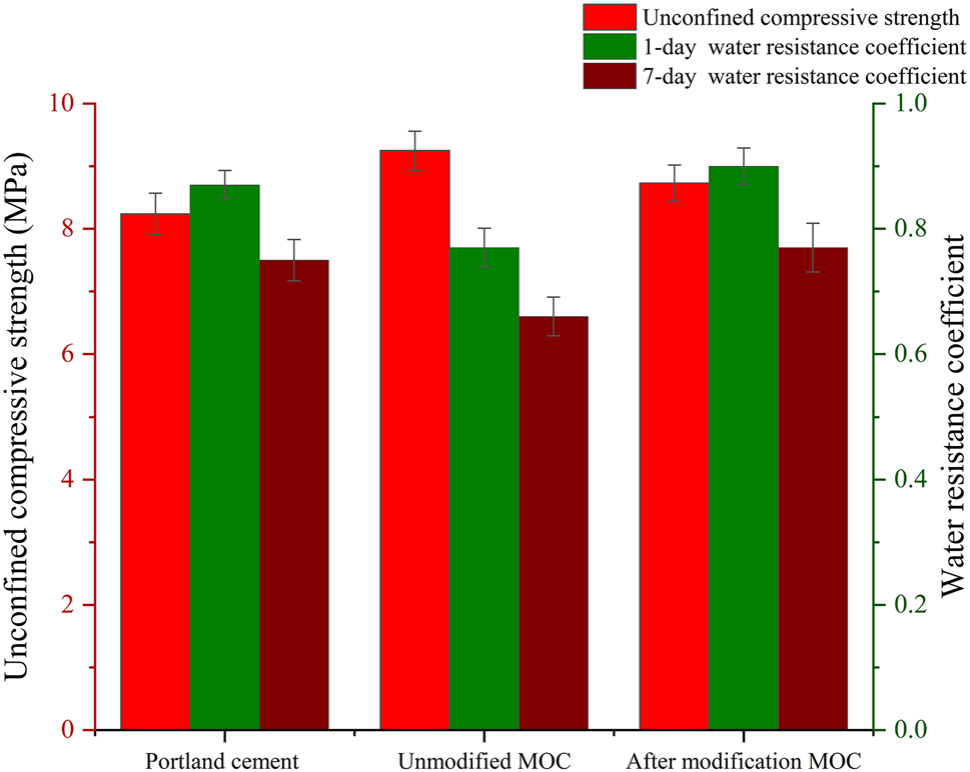
Verification of Final Strength and Water Resistance.

The 28-day unconfined compressive strength of the unmodified MOC-stabilized crushed stone was the highest, reaching 9.2 MPa (Fig. 13). However, the 1-day and 7-day water immersion resistance coefficients are only 0.78 and 0.66, respectively, which are much lower than that of the 5% cement stabilized crushed stone, which are 0.90 and 0.75. This demonstrates the insufficient water resistance of MOC-stabilized crushed stone and the necessity for its modification. After modification with citric acid and silica fume, the 28-day unconfined compressive strength and the 1-day and 7-day water resistance coefficients of the MOC-stabilized crushed stone were higher than the final strength and respective water immersion resistance coefficients of the 5% cement-stabilized crushed stone. This indicates the feasibility of using the citric acid and silica fume-modified MOC-stabilized crushed stone as the road base. It satisfies the load-bearing capacity and water-resistance performance requirements of the road base and demonstrates long-term reliability in terms of the water resistance.

## Conclusions

In this study, we aimed to determine a suitable mixture composition design for MOC-stabilized crushed stone to satisfy the requirements of road base materials. The findings of this study are summarized as follows:

i. The strength growth rate of the MOC-stabilized crushed stone was rapid within the first 4 days, which subsequently slowed down and leveled off. After 28 days, no significant increase in the strength was observed, indicating that the final strength must be determined based on the 28-day curing period.
ii. When applying the MOC-stabilized crushed stone to road bases, both the strength and water resistance performance requirements must be considered for practical engineering applications. The design of the mixing composition must adopt the 4-day unconfined compressive strength and 1-day water resistance coefficient as the technical indicators.
iii. A mixed composition design method was established for the MOC-stabilized crushed stone based on the RSM, by introducing a water resistance test and determining the water resistance coefficient of the material.
iv. The optimal mixing composition for using the MOC-stabilized crushed stone in a road base comprised 4.27% MOC and a MgO/MgCl_2_ molar ratio of 5.85. The best modification method involves substituting MOC with equal amounts of 1% citric acid and 10% silica fume, which can significantly enhance the water-resistance performance of the MCO-stabilized crushed stone, while ensuring that its strength remains essentially unaffected.
v. The feasibility of using MOC-stabilized crushed stone modified with citric acid and silica fume as a road base is clearly demonstrated. It satisfies the strength and water-resistance performance requirements for practical road-based engineering applications. However, the simultaneous use of two modifiers enhances the difficulty of construction to a certain extent.

## Data Availability

All data generated or analysed during this study are included in this published article.
